# Polymorphism of R353Q (rs6046) in factor VII and the risk of myocardial infarction: A systematic review and meta-analysis

**DOI:** 10.1097/MD.0000000000012566

**Published:** 2018-09-28

**Authors:** Haoming Huang, Wenjie Long, Weixuan Zhao, Ling Zou, Yudi Song, Junling Zuo, Zhongqi Yang

**Affiliations:** a The First Clinical Medical College, Guangzhou University of Chinese Medicine; b Department of Emergency; c Department of Geriatrics, The First Affiliated Hospital of Guangzhou University of Chinese Medicine, Guangzhou, P.R. China.

**Keywords:** *FVII* gene, myocardial infarction, R353Q, rs6046, single nucleotide polymorphism

## Abstract

**Objective:**

Genetic components substantially contribute to the development of myocardial infarction (MI), and R353Q polymorphism (rs6046) in *FVII* gene has been suspected to be associated with the risk of MI.

**Methods:**

A meta-analysis was conducted on the links between R353Q polymorphism and the susceptibility of MI. A comprehensive literature search was performed on 8 electronic databases. The main effects of the genotypes were estimated using a logistic regression approach. The odds ratios with 95% confidence intervals were calculated using the conventional summary method meta-analysis. The possible sources of heterogeneity among the included studies were explored using meta-regression analysis and subgroup analysis.

**Results:**

A total of 18 eligible case-control studies, comprising of 4701 cases and 5329 controls, were included. No overall statistical relationship was identified between R353Q and MI by any of the genetic models. The meta-regression demonstrated that the Asian population, body mass index (BMI) category, and diabetes affected the heterogeneity. In addition, subgroup analyses showed that heterogeneities were identified in Asian population and BMI category, which highly agree with the results of meta-regression.

**Conclusions:**

The current meta-analysis suggested that R353Q polymorphism was not associated with the MI risk. Asian population, BMI category, and diabetes might be related to the incidence of MI. However, large-scale, case-control studies with rigorous designs are essential to provide accurate evidence.

## Abstract 2

Supplemental Digital Content is available in the text

## 1 Introduction

Myocardial infarction (MI), commonly referred to as “heart attack,” occurs due to obstructions in the coronary arteries that diminish blood supply to the myocardium, causing rapid myocyte death. Acute changes in unstable atherosclerotic plaque underlie the primary cause of MI. Further initiation of coagulation pathway increases the thrombus bulks in the coronary arteries, which, eventually triggers MI. The coagulation factor VII (FVII), as an initiator of the extrinsic coagulation pathway, has been found to be correlated to the MI risk. Activated FVII binds to tissue factor, thereby activating the extrinsic coagulation, which promotes fibrin conversion and thrombosis, and leads to a blood clot in the vessels. This process even accelerates in the presence of unstable atherosclerotic plaques. Therefore, FVII levels are considered predictive of MI^[1,2]^ and are influenced by multiple factors, such as genetic architecture.^[3,4]^

In the 15-kb molecular genomic region surrounding the *FVII* gene, approximate 49 single-nucleotide polymorphisms (SNPs) were identified, of which, 4/7 functional variants were manifested to exert a regulatory control on circulating FVII levels.^[5]^ The R353Q polymorphism has been identified in exon 8 of the *FVII* gene that could up/downregulate the gene expression level, which was closely linked to the cardiovascular disease (CVD).^[6]^ Since guanine is substituted with adenine at the 353rd codon of the *FVII* gene, R353Q polymorphism is related to the missense replacement of the amino acid arginine (R) by glutamine (Q), which accounts for >20% of the variance at the FVII levels.^[7]^ In addition, the genetic variation was associated with R353Q polymorphism contributing to 30% of the variance in FVII coagulation activities and 23% of that in the FVII antigen.^[8]^ Patients with the RR genotype had a higher concentration of FVII than those with the RQ genotype, which, in turn, had a higher FVII concentration than those with the QQ genotype.^[8]^ The appropriate concentration and functionality of FVII might strike a balance between the cardiac protection and thrombosis, with the R allele favoring the latter.^[9]^ Thus, it is biologically plausible that R353Q polymorphism is involved in the thrombotic events, especially in the cases of MI.

Hitherto, clinical evidence focusing on the correlation between R353Q polymorphism and MI has been demonstrated worldwide. However, the observed associations of the studies were inconclusive. To resolve the conflicting results and test the above hypothesis, we conducted a systematic review and meta-analysis to investigate whether R353Q polymorphism was associated with the MI risk.

## 2 Methods

The current review demonstrated the association between R353Q polymorphism in *FVII* gene and MI risk. The meta-analysis was conducted according to Preferred Reporting Items for Systematic Reviews and Meta-Analyses Guidelines^[10]^ and the published research protocol on PROSPERO (CRD: 42017065196). This study is a meta-analysis that was conducted based on previously published studies; thus, no ethnical approval and patient consent are required.

### 2.1 Search strategies

The literature search was performed on the following electronic databases from their inception to May 2017 without language restriction: Medline, Embase, Cochrane Library, Web of Science, China National Knowledge Infrastructure (CNKI), Wanfang database, VIP database, and the China Biology Medicine (CBM) database. The search terms were as follow: (“myocardial infarction,” “heart infarction,” and “cardiovascular stroke”), (“coagulation factor VII,” “factor VII,” “F7,” “stable factor,” and “proconvertin”), (“genetic polymorphism,” “genetic variant,” “gene mutation,” “single nucleotide polymorphism,” and “SNP”). Titles and abstracts were examined by 2 authors independently for potentially eligible studies. Full-text articles were further reviewed to determine whether they conformed to the eligibility criteria and could be included/excluded in the final analysis. The retrieved references in the original publications were also scanned for additional relevant studies. Contradictory opinions were discussed to achieve a consensus.

### 2.2 Eligibility criteria

Studies included in this meta-analysis fulfilled the following inclusion criteria: (1) assessment of the association between R353Q polymorphism and MI; (2) studies conducted on human beings; (3) case-control design; (4) the data provided in the articles concerning the genotype frequencies should be sufficient to estimate the odds ratios (ORs) with the corresponding 95% confidence intervals (CIs) in both the case and control groups; and (5) the control group comprised healthy individuals, free of CVDs, and any relevant family history. Only the most recent publication was preserved in the final inclusions after the studies on the same population or duplication of previously published data were excluded. Moreover, studies with MI subjects were allocated into a subgroup under cases; these would be excluded in this meta-analysis unless the details were provided. Case reports, letters, reviews, editorials, and article comments were not suitable for meta-analysis, and thus, were excluded. In addition, we excluded the family-based studies and those conducted on autopsies or the participants with underlying organ dysfunctions.

### 2.3 Quality assessment

Two authors independently assessed the methodological quality of the selected studies using the Newcastle-Ottawa quality assessment scale (NOS) assessment tool.^[11]^ Studies with NOS scores ≥8 were considered as “high” quality, those with NOS scores ≤7 and ≥6 were classified as “medium” quality, and those with NOS scores ≤6 were considered as “low” quality and should be excluded from the final analysis. If the case of any disagreement regarding the quality assessment, consensuses were achieved by discussion or consulting a superior author in the research team.

### 2.4 Data extraction

Two authors independently conducted data extraction using a predesigned form including the following elements: (1) first author's name, publication year; (2) study region, ethnicity, and sample size; (3) participants’ characteristics including age, body mass index (BMI), hypertension, diabetes, and smoking status; (4) genotyping methods; and (5) number of cases and controls, and genotype frequency in cases and controls for R353Q polymorphism. The discrepancies between the 2 datasets were resolved by referring to the original articles, and uncertainties were adjudicated by a superior author.

### 2.5 Data analysis

Fisher exact test was used to assess the deviation from Hardy-Weinberg equilibrium in controls for each study, and *P* < .01 was considered as significant disequilibrium. Heterogeneity among the studies was confirmed by χ^2^-based Cochran Q statistic at a significance level of *P* ≤ .10. The *I*-squared (*I*^2^) metric >50% also served as an evidence of significant inconsistency between studies. If no heterogeneity were identified, a fixed effects logistic regression approach would be used to assess the primary effect of the genotype.^[12,13]^ Presuming that the genotype effects were identical across studies and that the genotypes and studies were considered as fixed effects, the logistic regression models were defined as^[14]^:
 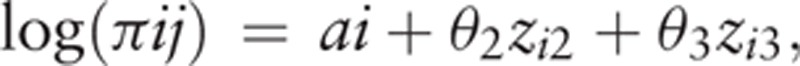


where *π*_*ij*_ was denoted the underlying risk of a person with *j*th genotype in the *i*th study; *a*_*i*_ was the indicator of the study-specific fixed effects. Since R allele was considered the risk allele, reportedly increasing the risk of MI, we selected the QQ genotype as the reference category and created mock variables *z*_*i*2_ and *z*_*i*3_ for RQ and RR, respectively. Parameters *θ*_2_ and *θ*_3_ were logOR_RQ/QQ_ and logOR_RR/QQ_, respectively. If there were heterogeneity on either of the ORs, study-specific random coefficients *ν*_*ij*_ would be incorporated into the above model:
 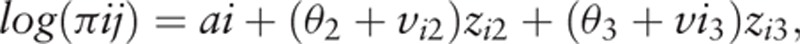


Thus, a random-effects logistic regression would be used to calculate the effects. Henceforth, the most plausible genetic model was determined by interpreting the relationship of parameters *θ*_2_ and *θ*_3_^[13]^: (1) *θ*_2_ = *θ*_3_ = 0 suggested no significant genetic association; (2) *θ*_2_ = 0 and *θ*_3_ > 0 suggested a recessive model; (3) *θ*_2_ = *θ*_3_ > 0 suggested a dominant model; (4) *θ*_3_ > *θ*_2_ > 0, a codominant model; (5) 2*θ*_3_ = *θ*_2_, an additive model was appropriated. Crude ORs of the genetic models, identified from the logistic regression, would be pooled using the conventional summary method for meta-analysis. However, if the logistic regression did not infer any molecular relationship between the genotype and the event; crude ORs for all genetic models were pooled to obtain a comprehensive assessment of the associations. The 3 genotypes would collapse into 2 groups according to the genetic models: allele model (R vs Q), homozygote model (RR vs QQ), heterozygote model (RQ vs QQ), additive model (RR vs RQ), dominant model (RR + RQ vs QQ), recessive model (RR vs RQ + QQ), and codominant model (RQ vs RR + QQ). The fixed effects model (the Mantel-Haenszel method) was used to pool the ORs in the absence of inconsistency. On the contrary, the random effects model (DerSimonian and Laird method) would be adopted in the presence of significant heterogeneity. Mixed-effects meta-regression analysis with the Knapp-Hartung modification^[15]^ was also performed to identify the source of heterogeneity and the model was fitted via restricted maximum-likelihood estimation. In addition to heterogeneity test, subgroup analyses were performed according to ethnicity, age, BMI, sex, and study quality to explore the potential sources of heterogeneity among studies. Excluding the studies with zero cell count or all events would potentially create the risk of inflating the magnitude of the pooled effects,^[16]^ 0.5 is added for continuity correction and singularity prevention. Statistical analyses were performed using R software 3.4.0,^[17]^ summary method meta-analyses were conducted using the “meta” package version 4.8-2^[18]^ and meta-regression analyses were conducted with the “metafor” package version 2.0-0^[19]^ in R.

### 2.6 Sensitivity analysis

Sensitivity analysis would be employed to evaluate the stability of the results and the potential origins of heterogeneity. A new analysis would be performed by excluding the included studies sequentially to examine the influences on the combined ORs.

### 2.7 Publication bias

The potential publication bias was assessed by the graphical inspection of the asymmetry of the Begg funnel plot and statistically evaluated through Begg rank correlation^[20]^ and Egger linear regression test.^[21]^ Furthermore, to assess the “Proteus” phenomenon,^[22]^ the tendency of conflict of early findings with the initial conclusion as a consequence of publication bias and cumulative meta-analysis was evaluated.

## 3 Results

### 3.1 Characteristics of studies

**Figure 1:**
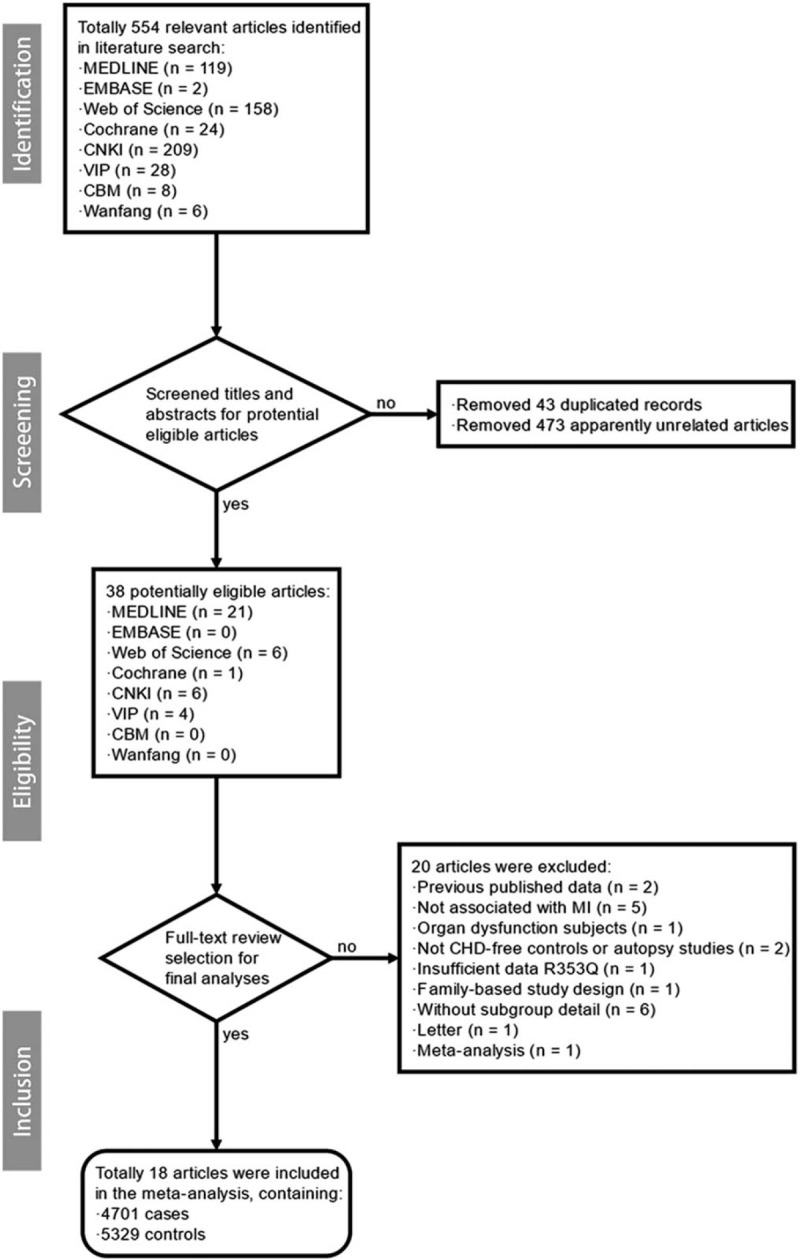
Flow diagram of the study selection process. CBM = China Biology Medicine, CHD = chronic heart disease, CNKI = China National Knowledge Infrastructure, n = number of the studies.

Figure 1 illustrates the selection process of the studies. A total of 18 studies,^[6,23–39]^ which satisfied the eligibility criteria, were identified from 8 electronic databases (Medline, Embase, Cochrane Library, Web of Science, CNKI, VIP, Wanfang, and CBM). The characteristics of the included studies are summarized in Table 1, whereas the reasons for the excluded studies are recorded in Supplementary Table S1. The included studies were published between 1999 and 2014. Nine studies^[23–26,29,31–33,35]^ comprised the Caucasian population, whereas 6^[6,27,30,36–38]^ constituted of Asians. On the contrary, 3 studies,^[28,34,39]^ performed in multiethnic areas (US,^[28]^ Costa Rica,^[34]^ and Mexico^[39]^), did not provide any information about the ethnicity in the original articles, and thus, were categorized as “Other.” Notably, Lane et al^[24]^ conducted the study in 4 different regions in Europe, and the results were reported separately. Therefore, these 4 studies were included independently in the analyses. The R353Q polymorphism was detected by polymerase chain reaction-restriction fragment length polymorphism (PCR-RFLP) in all the included studies. The study quality was assessed by NOS; 4 of the included studies^[24,29,33,38]^ were assessed to be “high” quality, whereas the remaining 14 studies^[6,23,25–28,30–32,34–37,39]^ were assessed to be “medium” quality. A total of 18 studies containing 4701 cases and 5329 controls were included in this meta-analysis. Table 2 outlines the genotype frequencies of the studies. All the studies were in agreement with Hardy-Weinberg equilibrium at a significance level of *P* > .01.

**Table 1:**
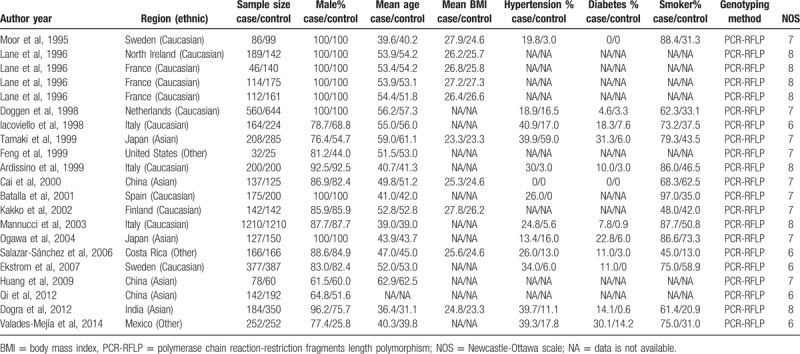
Basic characteristics of studies included in the meta-analysis.

**Table 2:**
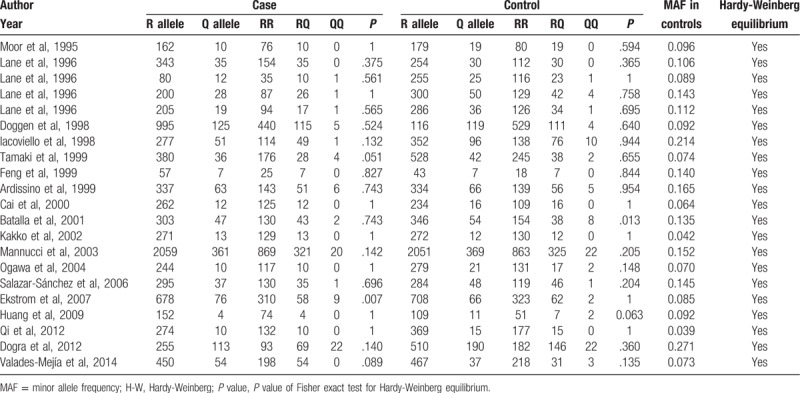
Frequencies of the genotype of eligible studies in the meta-analysis.

### 3.2 Association between FVII R353Q polymorphism and MI

**Figure 2:**
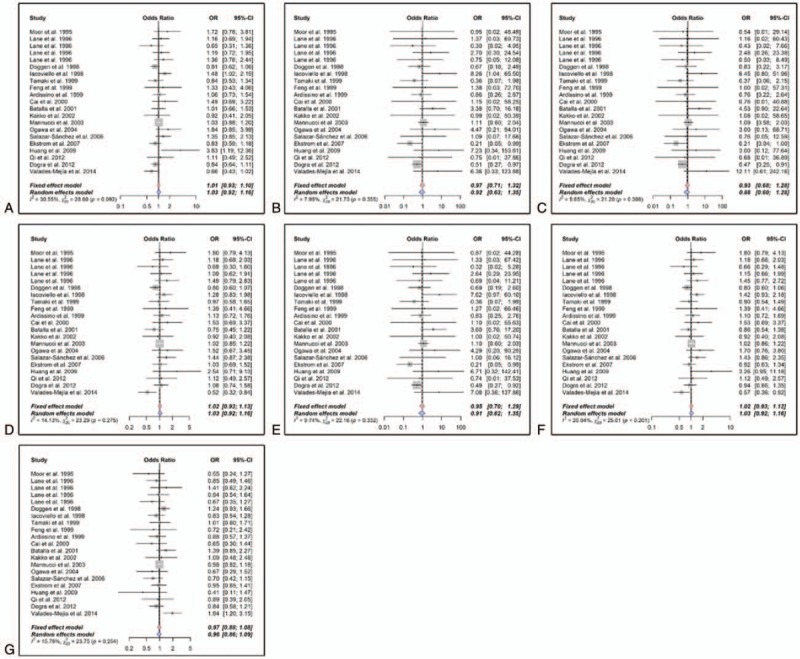
Forests for R353Q polymorphism (rs6046) and myocardial infarction MI risk. A, Allele model (R vs Q). B, Homozygote model (RR vs QQ). C, Heterozygote model (RQ vs QQ). D, Additive model (RR vs RQ). E, Dominant model (RR + RQ vs QQ). F, Recessive model (RR vs RQ + QQ). G, Codominant model (RR + QQ vs RQ). Vertical and horizontal lines represent ORs and the corresponding 95% CIs of each study. Areas of gray square stand for the studies-specific weight. Red and blue stroked diamonds represent the pooled ORs and 95% CIs of the overall population with fixed effect and random effects model, respectively. 95% CI = 95% confidence interval,
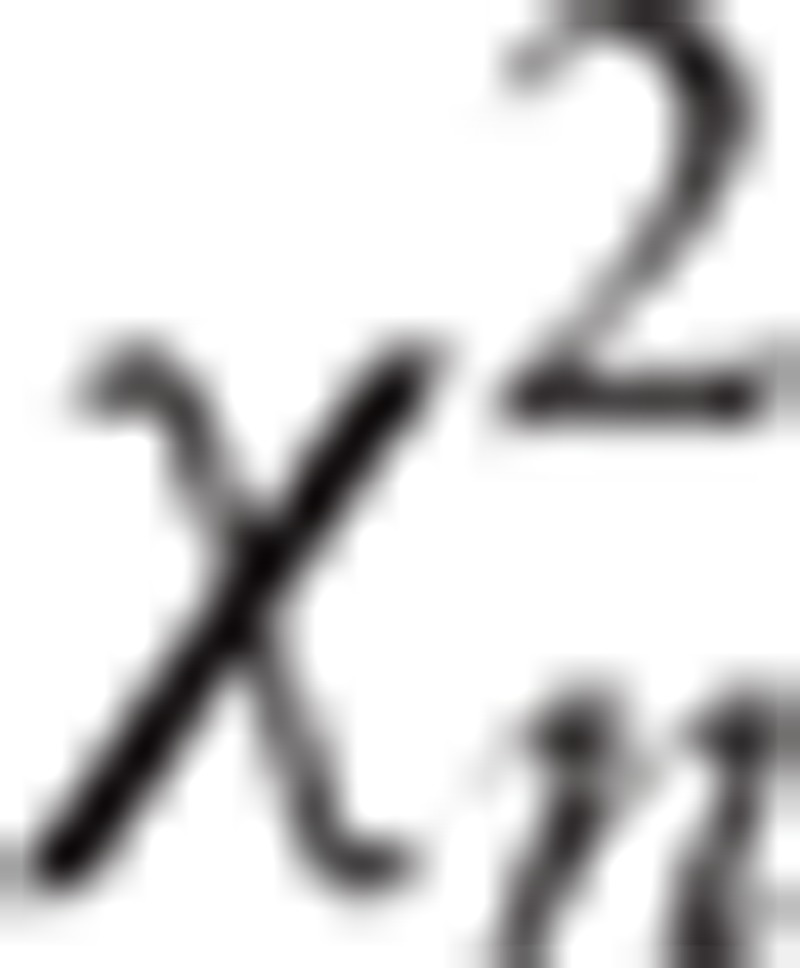
 = heterogeneity Q statistic, *I*^2^ = *I*-squared metric of the heterogeneity, n = degrees of freedom; OR = odds ratio; *P* = *P* value of heterogeneity Q test.

Heterogeneity Q test of OR_RQ/QQ_ and OR_RR/QQ_ were *P*_H_ = .385 and *P*_H_ = .355, respectively. A fixed effect logistic regression model was used to estimate the primary effects of the genotype, and the pooled OR_RQ/QQ_ and OR_RR/QQ_ were 0.945 (95% CI: 0.683–1.309, *P*_sig_ = .736) and 0.97 (95% CI: 0.70–1.33, *P*_sig_ = .842), respectively. Parameters *θ*_2_ and *θ*_3_ in the logistic regression were −0.06 (95% CI: −0.38–0.27) and −0.03 (95% CI: −0.35–0.29), respectively. Wald test was used to compare these 2 parameters, which indicated that *θ*_2_ = *θ*_2_ = 0 (*P* = .673), and R353Q polymorphism might be unrelated to the risk of MI. A likelihood ratio test was also performed to draw a comparison between the models with and without the genotype (*P* = .869). Since no plausible genetic model could be deduced under logistic regression, a conventional summary method meta-analysis was carried out to calculate the crude ORs. The results of the meta-analysis are summarized in Table 3, and the forest plots are shown in Figure 2. These studies were in agreement in all the genetic models as reviewed in heterogeneity Q test (*P*_H_ > .1) and *I*^2^ metric (*I*^2^ < 50%) except the allele model (*P*_H_ = .092). No significant correlation (*P*_sig_ for *z* test > .05) was identified between R353Q polymorphism and MI risk with pooled ORs of 1.03 (95% CI: 0.92–1.16) in the allele model, 0.97 (95% CI: 0.71–1.32) in the homozygote model, 0.93 (95% CI: 0.68–1.28) in the heterozygote model, 1.02 (95% CI: 0.93–1.13) in the additive model, 0.95 (95% CI: 0.7–1.29) in the dominant model, 1.02 (95% CI: 0.93–1.12) in the recessive model, and 0.97 (95% CI: 0.88–1.08) in the codominant model.

**Table 3:**
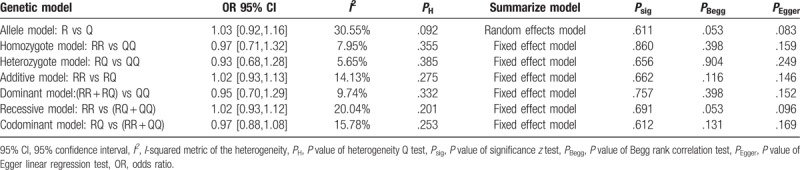
Main results of the meta-analysis.

### 3.3 Heterogeneity analyses

We subsequently evaluated the potential influence of study characteristics on the results via meta-regression analysis and subgroup analysis. Results of the meta-regression and subgroup analyses are summarized in Tables 4 and 5, respectively. Heterogeneity across studies in terms of study characteristics is examined, for instance ethnicities, average age, BMI category, male candidates, hypertension patients, diabetes, and cigarette consumers. Heterogeneity analyses were also carried out to test the potential influence of the studies’ quality.

**Table 4:**
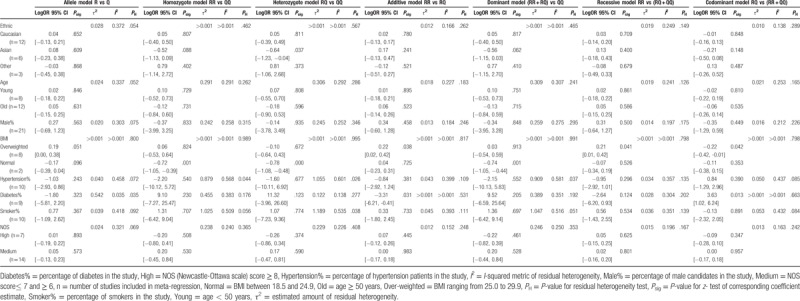
Summary of meta-regression analysis.

**Table 5:**
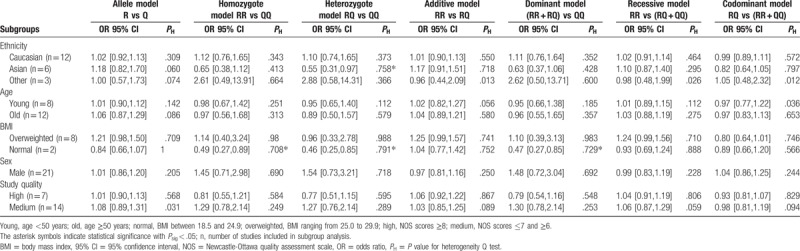
Subgroup analysis on ethnicity, age, body mass index, and sex.

In meta-regression analyses, heterogeneity was detected under the heterozygote model among Asian population (LogOR = −0.64, 95% CI: −1.23 – −0.04, *P*_sig_ = .037). Moreover, heterogeneities were observed in normal BMI group under homozygote, heterozygote, and dominant models (*P*_sig_ < .05). Although overweighted BMI significantly influenced the effect sizes for additive, recessive, codominant models (*P*_sig_ < .05). Diabetes might also contribute to the heterogeneity under homozygote (*P*_sig_ = .031), and codominant models (*P*_sig_ = .013). Other variables such as mean age, male population, hypertension patients, cigarette consumers, and study quality were not significantly associated with the effect size.

In subgroup analyses, heterogeneities were identified in Asian population and BMI, which highly agree with the results of meta-regression. No significant association with the MI risk was found in subgroup analyses among age, male population, and study quality.

### 3.4 Sensitivity analyses and assessment of publication bias

**Figure 3:**
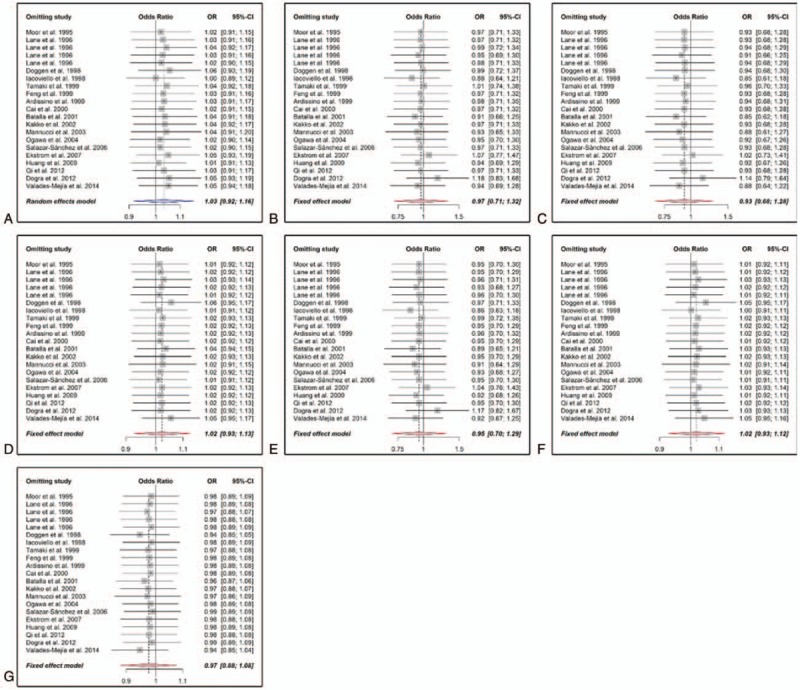
Influence plots of the included studies. A, Allele model (R vs Q). B, Homozygote model (RR vs QQ). C, Heterozygote model (RQ vs QQ). D, Additive model (RR vs RQ). E, Dominant model (RR + RQ vs QQ). F, Recessive model (RR vs RQ + QQ). G, Codominant model (RR + QQ vs RQ). Vertical and horizontal lines represent ORs and 95% CIs pooled by successively excluding one study in turn. Red stroked diamonds represent the overall estimates (pooled ORs and 95% CIs) of population with fixed effect model, and blue stroked diamonds symbolize results pooled with random effects model. 95% CI = 95% confidence interval, OR = odds ratio.

**Figure 4:**
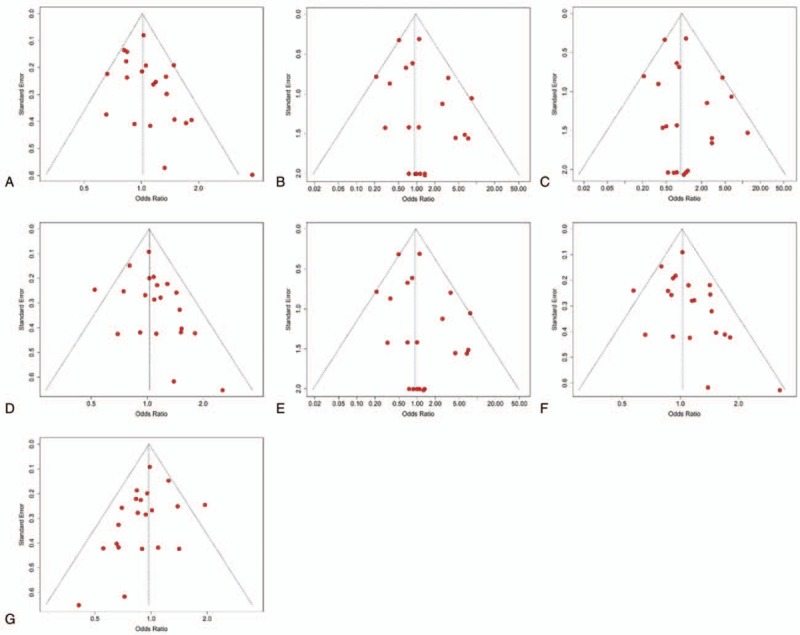
Funnel plots of the included studies. A, Allele model (R vs Q). B, Homozygote model (RR vs QQ). C, Heterozygote model (RQ vs QQ). D, Additive model (RR vs RQ). E, Dominant model (RR + RQ vs QQ). F, Recessive model (RR vs RQ + QQ). G, Codominant model (RR + QQ vs RQ). X- and Y-axes of the plots stand for odds ratios of each study and standard errors of the genetic effect estimates, respectively. Red solid circles represent separate study. The horizontal dashed lines represent the pooled odds ratios.

**Figure 5:**
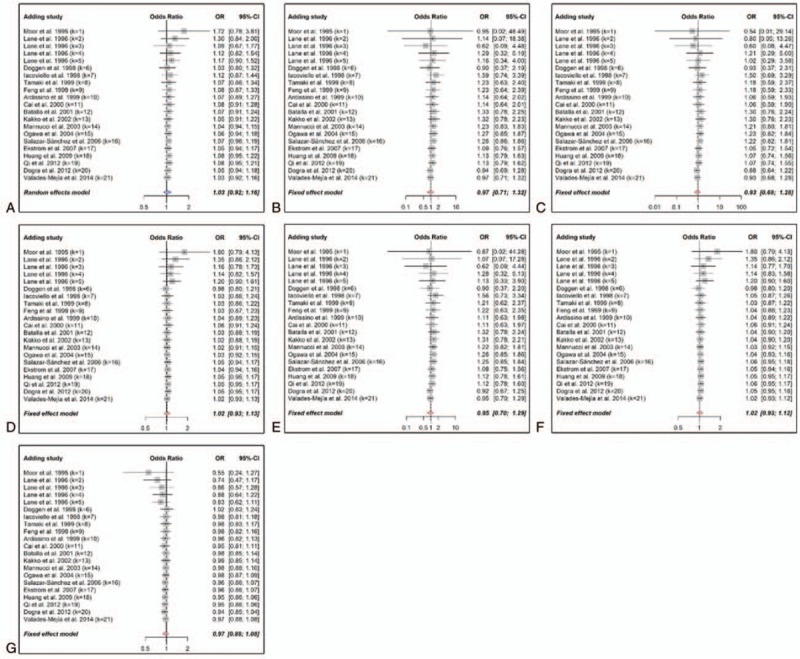
Cumulative forests for R353Q polymorphism (rs6046) and myocardial infarction (MI) risk. A, Allele model (R vs Q). B, Homozygote model (RR vs QQ). **C**, Heterozygote model (RQ vs QQ). D, Additive model (RR vs RQ). E, Dominant model (RR + RQ vs QQ). F, Recessive model (RR vs RQ + QQ). G, Codominant model (RR + QQ vs RQ). Vertical and horizontal lines represent pooled ORs and 95% CIs by adding studies serially. Red stroked diamonds represent the overall pooled ORs and 95% CIs with fixed effect model, and blue stroked diamonds symbolize results pooled with random effects model. The *k* after each study label stands for the number of studies that were included for the result. CI = confidence interval, OR = odds ratio.

We evaluated the influence of each study on the overall estimates in this meta-analysis by sensitivity analysis such that any single study could not impact the outcomes considerably. The results were stable as found by sensitivity analyses, and no single study was responsible for the pooled ORs (Fig. 3). The selectivity of publication could lead to bias in the conclusions, which might be contradictory to the objective of the current meta-analysis. Begg funnel plots are shown in Figure 4, and results of Begg and Egger tests are recorded in Table 3. No potential publication bias was identified via either of the genetic models, indicating that the outcomes were stable and robust. A cumulative meta-analysis was also carried out to assess the “Proteus” phenomenon, a potential source of publication bias. The results suggested that all the studies were consistently associated with the final results, and no study was expected to differ from the pooled ORs (Fig. 5).

## 4 Discussion

Coagulation FVII is one of the serine proteases involved in thrombotic formation. High levels of FVII are associated with increased risk of MI. Conversely, low levels of FVII are protective against MI. The genetic variants in the *FVII* gene have been widely studied. The R allele in R353Q polymorphism was suspected of raising the risk of MI.^[26]^ Whereas, the Q allele was found to be related to the impairments of FVII production according to the in vitro and in vivo studies, thereby accounting for the reduction in plasma FVII levels.^[40]^

Although strong clinical associations have been confirmed between R353Q polymorphism and FVII levels,^[40,41]^ the influence of these association on the clinical outcomes remains controversial. Several meta-analyses have explored the relationship of R353Q polymorphism and CVD predisposition, aspiring much controversy. Wu and Tsongalis^[9]^ found that Q allele of R353Q polymorphism was correlated with the reduced risk of CVD, whereas, Ye et al^[42]^ reported that the polymorphism had no significant overall association with CVD. On the contrary, Mo et al^[43]^ identified a significant correlation between R353Q polymorphism and CVD in the Asian population; the Q allele was reported as a protective factor. However, the extent to which the R353Q polymorphism contributed to the risk of MI remains to be explored. To the best of our knowledge, the current study is the first meta-analysis emphasizing the genetic effects of R353Q polymorphism on MI.

In the present meta-analysis, we quantitatively assessed the association between the R353Q polymorphism in the *FVII* gene and the susceptibility of MI. Thus, we selected 18 eligible studies including 4701 cases and 5329 controls in this meta-analysis. Without prior knowledge about the genetic model, we estimated the effects of the molecular association by a logistic regression method, which was speculated to reduce the erroneous interpretation of the combined results and avoid unnecessary comparisons.^[13]^ In the logistic regression analysis, no possible genetic model was inferred (*θ*_2_ = *θ*_3_ = 0), which indicated that the R353Q polymorphism might not associate with MI. Subsequently, for a comprehensive inspection of the genetic effects, crude ORs and corresponding 95% CIs were calculated using the conventional summary methods on allele, homozygote, heterozygote, additive, dominant, recessive, and codominant models, respectively. Nevertheless, according to the current results, R353Q polymorphism was neither a favorable nor a contrary indicator of MI under all the genetic models.

Ethnicity is a complex phenomenon constructed of different biology, history, cultural orientations, and practices^[44]^ that affect the predisposition of diseases. Also, the distributions of FVII polymorphisms vary across different ethnicities, which might contribute to various clinical outcomes.^[45–47]^ Thus, ethnicity is a critical stratification in the current meta-analysis. Subgroup analysis and meta-regression analysis were performed on “Caucasian,” “Asian,” and “Other” ethnic populations. A significantly decreased risk of MI was observed in the heterozygote model (RQ vs QQ) among Asians, whereas the no association was noted in Caucasians and other ethnic populations. However, these results were not in agreement with the previous study, wherein the Q allele significantly reduced the MI risk in Asians.^[43]^

Obesity is one of the traditional nongenetic risk factors that increase the susceptibility of MI, ischemic stroke, and diabetes mellitus.^[48–50]^ Reportedly, the FVII levels are positively associated with carotid intima-media thickness (subclinical atherosclerosis manifestation),^[51]^ and the strength of the association could be modified by BMI.^[52]^ Moreover, the association between FVII polymorphisms and the MI susceptibility could be partially mediated through the interaction with BMI.^[53]^ According to our results, stratification of BMI might partially explain the heterogeneity across outcomes under homozygote, heterozygote, additive, dominant, recessive, and codominant models. However, owing to the relatively small sample size, there could be potential risks of errors in the estimations. The mechanism underlying the FVII genetic architecture affecting the risk of MI appears to be complicated. Similar to other risk factors, BMI changes over the lifetime, which might affect the FVII genotype-phenotype expression.

The hemostatic system varies with elderly, hypertension, diabetes, and cigarette consumption, which trend to thromboembolism events.^[54–58]^ According to our results, diabetes might have an influence on the MI risks. However, the remaining results from the heterogeneity analyses on those factors did not show any statistical association under all genetic models. Furthermore, study quality and sex were not potential sources of heterogeneity.

In the current study, SNP was genotyped by PCR-RFLP. Thus, no inconsistency was noted in the genotyping method. However, similar to the other enzymatic approaches that were used previously to detect the SNPs, this approach presented some drawbacks that could not be circumvented, such as low throughput, low specificity, and disregard for molecular interactions. With the advancement in the technology, hybridization-based strategies (TaqMan probe, microarray, and invader assay), mass spectrometry approaches, and sequencing tools were developed and proposed for an accurate and efficient detection of the SNPs.^[59]^ Incorporating with the genome-wide association study design, the whole genome can be readily screened to investigate the genetic interactions and genotype-phenotype associations.^[60]^

The sensitivity analysis suggested that any single study could not influence the combined results. No publication bias was detected under all genetic models, and cumulative meta-analyses deduced that none of the studies were expected to be different from the pooled ORs. Thus, the results withstood the test of stability and reliability. Nevertheless, the present study had some potential limitations. First, the current results are based on unadjusted estimates. Thus, a precise analysis should be conducted using individual patient data, which would allow researchers to adjust for covariates, including patients’ conditions, lifestyle, family history, and environmental factors. Second, only published studies were included, even though we did not detect a potential bias in this meta-analysis. Third, a relatively small sample size might be not sufficient for conclusive results.

In summary, the current results suggested that R353Q polymorphism was not associated with the risk of MI. However, we identified a significantly reduced MI risk in Asians; additionally, BMI category, and diabetes might also affect the incidence of MI. These warranted further investigations about the effects of gene-environment interactions on the MI risk. Moreover, well-designed studies with larger sample size are required to substantiate the findings in this meta-analysis.

## Acknowledgments

The authors are grateful to Professor Licheng Zhao from Guangzhou University of Chinese Medicine for his inspiring guidance to this work, and the platform provided from Lingnan Medical Research Center to ensure the successful completion of this work. The authors also thanks to Miss Jingyi Xu for her help in data entry and quality management.

## Author contributions

H. Huang and W. Long performed data analyses and wrote the main manuscript. L. Zou and Y. Song involved in search and selection of the eligible articles and data collection. W. Zhao was responsible for the figures preparation. J. Zuo and Z. Yang designed the research study. All the authors reviewed and approved the manuscript.

**Funding acquisition:** Junling Zuo, Zhongqi Yang.

**Investigation:** Wenjie Long, Weixuan Zhao, Ling Zou, Yudi Song.

**Methodology:** Haoming Huang, Wenjie Long, Weixuan Zhao, Ling Zou, Yudi Song, Junling Zuo, Zhongqi Yang.

**Project administration:** Haoming Huang.

**Resources:** Haoming Huang, Wenjie Long.

**Validation:** Wenjie Long.

**Visualization:** Haoming Huang.

**Writing – original draft:** Haoming Huang, Wenjie Long, Junling Zuo, Zhongqi Yang.

**Writing – review and editing:** Haoming Huang, Wenjie Long, Weixuan Zhao, Ling Zou, Yudi Song, Junling Zuo, Zhongqi Yang.

## Supplementary Material

Supplemental Digital Content
