# Supplementary material for: Polymorphism of R353Q (rs6046) in factor VII and the risk of myocardial infarction: A systematic review and meta-analysis
Source: Medicine (Baltimore). 2018 Sep 28;97(39):e12566. doi: 10.1097/MD.0000000000012566 (PMC6181591; doi:10.1097/MD.0000000000012566)
Supplement: Supplemental Digital Content [file medi-97-e12566-s001.docx]

**Polymorphism of R353Q (rs6046) in factor VII and the risk of myocardial infarction: A systematic review and meta-analysis.**

Haoming Huang^1,§^, Wenjie Long^1,§^, Weixuan Zhao^1^, Ling Zou^1^, Yudi Song^1^, Junling Zuo^2,^**^*^**, and Zhongqi Yang^3,^**^*^**

^§^These authors are co-first authors and contributed equally to this work

^1^ No. 1 Clinical College, Guangzhou University of Chinese Medicine, No.12 Jichang Road, Baiyun District, Guangzhou, 510405, P.R. China

^2^ Department of Emergency, No. 1 Affiliated Hospital of Guangzhou University of Chinese Medicine, No.16 Jichang Road, Baiyun District, Guangzhou, 510405, P.R. China

^3^ Department of Geriatrics, No. 1 Affiliated Hospital of Guangzhou University of Chinese Medicine, No.16 Jichang Road, Baiyun District, Guangzhou, 510405, P.R. China

# ^*^Corresponding authors

Zhongqi Yang,

Department of Geriatrics, No. 1 Affiliated Hospital of Guangzhou University of Chinese Medicine, No.16 Jichang Road, Baiyun District, Guangzhou, 510405, P.R. China

Tel: 13560231626

Fax: 86-20-36591595

E-mail: [yang_zhongqi@163.com](mailto:yang_zhongqi@163.com)

Junling Zuo,

Department of Emergency, No. 1 Affiliated Hospital of Guangzhou University of Chinese Medicine, No.16 Jichang Road, Baiyun District, Guangzhou, 510405, P.R. China

E-mail: [dr.zuo@163.com](mailto:dr.zuo@163.com)

**Running title:** Polymorphism of R353Q and the risk of MI

**Table S1: Details of the excluded studies and reasons of exclusion.**

| **Author** | **Publication year** | **Title** | **Exclusion reasons** |
| --- | --- | --- | --- |
| Cai et al. | 2000 | Association of coagulation factor VII with the risk of myocardial infarction in the Chinese. | Previously published data |
| Peyvandi et al. | 2000 | A novel polymorphism in intron 1a of the human factor VII gene (G73A): Study of a healthy Italian population and of 190 young survivors of myocardial infarction. | No sufficient data |
| Girelli et al. | 2000 | Polymorphisms in the factor VII gene and the risk of myocardial infarction in patients with coronary artery disease. | Not heathy controls |
| Tao et al. | 2000 | Analysis of the relationship between FVII activity and the polymorphism. | No subgroup detail |
| Mikkelsson et al. | 2002 | Genetic variation in coagulation factors II, V, VII and fatal MI. | Letter |
| Kang et al. | 2002 | Study on plasma coagulation factor VII (FVII) levels and polymorphisms of FVII gene in patients with coronary heart disease. | No subgroup detail |
| Xu et al. | 2002 | Polymorphisms of the coagulation factor VII gene in patients with coronary heart disease and health volunteers. | No subgroup detail |
| Sha et al. | 2002 | Relationship between FVII gene polymorphism and coronary heart disease in Hui and Han populations in Ningxia. | No subgroup detail |
| Xu et al. | 2003 | Polymorphisms in the genes for coagulation factor II, V, VII in patients undergoing coronary angiography. | Previously published data |
| Xu et al. | 2003 | Association of coagulation factor V, VII gene polymorphisms with coronary heart disease. | No subgroup detail |
| Yeh et al. | 2004 | Prognosis of young ischemic stroke in Taiwan: Impact of prothrombotic genetic polymorphisms. | Not associated with MI |
| Lindman et al. | 2004 | Coagulation factor VII, R353Q polymorphism, and serum choline-containing phospholipids in males at high risk for coronary heart disease. | Not associated with MI |
| Pegoraro et al. | 2005 | Coagulation gene polymorphisms as risk factors for myocardial infarction in young Indian Asians. | Family based study design |
| Jeffery et al. | 2005 | A protective contribution of the Q allele of the R353Q polymorphism of the Factor VII gene in individuals with chronic stable angina? | Not associated with MI |
| Ye et al. | 2006 | Seven haemostatic gene polymorphisms in coronary disease: meta-analysis of 66,155 cases and 91,307 controls. | Meta-analysis |
| Fujimaki et al. | 2009 | Association of genetic variants with myocardial infarction in Japanese individuals with chronic kidney disease. | Organ dysfunction subjects |
| Huang et al. | 2009 | The coagulation factor VII gene polymorphisms in patients with myocardial infarction in Ningxia Hui and Han population. | No subgroup detail |
| Pushkov et al. | 2011 | Polymorphic markers Ala455Val of the THBD gene and Arg353Gln of the F7 gene and genetic association with unfavorable outcomes of coronary atherosclerosis in patients with a history of acute ischemic heart disease. | Not heathy controls |
| Boroumand et al. | 2013 | Association between R353Q polymorphism for coagulative factor VII and severity of coronary artery disease in Iranian population. | Not associated with MI |
| Kaur et al. | 2016 | Genetic polymorphisms, biochemical factors, and conventional risk factors in young and elderly North Indian patients with acute myocardial infarction. | Not associated with MI |

MI, myocardial infarction; CHD, coronary heart disease; THBD, Thrombomodulin.
